# Estimating excess mortality during heat waves using a causal inference framework and non-randomized time series

**DOI:** 10.64898/2026.10.13.26365467

**Published:** 2026-10-14

**Authors:** Yasmine Mabene, Lauren Mock, Luke Miratrix, Young Lee, Marie-Abèle Bind

## Abstract

Researchers must often rely on observational data to draw conclusions about the causal relationship between an exposure and an outcome. This is common in environmental epidemiology research, where the assignment to an exposure can be unethical or impossible. Consider a setting in which we seek to estimate excess mortality during heat waves, but are unable to randomize individuals to moderate or extreme heat. We propose to follow a causal framework for non-randomized time series that uses what we call *historical time series matching* to construct hypothetical randomized experiments, in which background covariates are balanced. We applied this framework and assessed the following causal question: Are daily mortality counts among elderly Americans higher during hot versus warm summer time periods? We addressed this causal question by drawing frequentist, Fisherian, and Bayesian inferences for five American cities. We estimated the average treatment effect of heat on three-day mortality with 95% Fisherian intervals to be 0.5 [-3.6, 4.4] deaths in Pittsburgh, 3.7 [0.7, 6.6] deaths in Seattle, 12.5 [6.1, 18.9] deaths in Chicago, 8.5 [1.4, 15.6] deaths in Los Angeles, and 20.3 [11.2, 29.5] deaths in New York.

